# Establishing a proactive safety and health risk management system in the fire service

**DOI:** 10.1186/s12889-015-1675-8

**Published:** 2015-04-19

**Authors:** Gerald S Poplin, Keshia M Pollack, Stephanie Griffin, Virginia Day-Nash, Wayne F Peate, Ed Nied, John Gulotta, Jefferey L Burgess

**Affiliations:** Center for Applied Biomechanics, University of Virginia, Charlottesville, USA; Mel & Enid Zuckerman College of Public Health, University of Arizona, Tucson, USA; Bloomberg School of Public Health, Johns Hopkins University, Baltimore, USA; Tucson Fire Department, Tucson, USA

**Keywords:** Risk management, Fire service, Safety and health

## Abstract

**Background:**

Formalized risk management (RM) is an internationally accepted process for reducing hazards in the workplace, with defined steps including hazard scoping, risk assessment, and implementation of controls, all within an iterative process. While required for all industry in the European Union and widely used elsewhere, the United States maintains a compliance-based regulatory structure, rather than one based on systematic, risk-based methodologies. Firefighting is a hazardous profession, with high injury, illness, and fatality rates compared with other occupations, and implementation of RM programs has the potential to greatly improve firefighter safety and health; however, no descriptions of RM implementation are in the peer-reviewed literature for the North American fire service.

**Methods:**

In this paper we describe the steps used to design and implement the RM process in a moderately-sized fire department, with particular focus on prioritizing and managing injury hazards during patient transport, fireground, and physical exercise procedures. Hazard scoping and formalized risk assessments are described, in addition to the identification of participatory-led injury control strategies. Process evaluation methods were conducted to primarily assess the feasibility of voluntarily instituting the RM approach within the fire service setting.

**Results:**

The RM process was well accepted by the fire department and led to development of 45 hazard specific-interventions. Qualitative data documenting the implementation of the RM process revealed that participants emphasized the: value of the RM process, especially the participatory bottom-up approach; usefulness of the RM process for breaking down tasks to identify potential risks; and potential of RM for reducing firefighter injury.

**Conclusions:**

As implemented, this risk-based approach used to identify and manage occupational hazards and risks was successful and is deemed feasible for U.S. (and other) fire services. While several barriers and challenges do exist in the implementation of any intervention such as this, recommendations for adopting the process are provided. Additional work will be performed to determine the effectiveness of select controls strategies that were implemented; however participants throughout the organizational structure perceived the RM process to be of high utility while researchers also found the process improved the awareness and engagement in actively enhancing worker safety and health.

**Electronic supplementary material:**

The online version of this article (doi:10.1186/s12889-015-1675-8) contains supplementary material, which is available to authorized users.

## Background

Risk management (RM) has been increasingly adopted within industry as a formal proactive approach to improving occupational safety and health. RM creates a structure for individual operations to develop solutions to the risks faced, based on the surrounding environment, conditions, equipment, and personnel involved. Though risk has been considered and quantified in numerous ways, the RM techniques described herein, most notably formal (structured) risk assessment, have their roots in the Nuclear Regulatory Commission during the 1970s [1]. Further risk-based regulations, incorporating the “duty of care” stance, stemmed from the 1972 United Kingdom (UK) parliamentary commission report for occupational safety and health prevention legislation [2], requiring that everything reasonably practical be done to protect the worker health and safety (by employers, employees, and any others that may influence workplace hazards). RM is currently most broadly implemented as part of the International Organization for Standardization (ISO) 31000 standard [3].

RM was initially focused on high-risk industries. During the mid-to-late 1980s, the Australian coal mining industry began to implement a risk-based approach to safety and health by government regulation; however, it wasn’t until two disasters – the Moura disaster of 1994, in which an underground explosion killed 11 miners, and the 1996 Gretley disaster, where 4 people drowned when tunneling into an old adjacent mine shaft that had not been documented in the mining plans – that meaningful changes to safety were initiated. In one of the only published studies to date on the effectiveness of these regulations on worker safety, adoption of RM in the Australian mining industry was associated with marked decreases in lost-time injuries, as compared with the United States (US), where injury prevention efforts in the workplace typically revolve around some type of compliance-based system, consistent with the major regulatory requirements [4].

**Figure 1:**
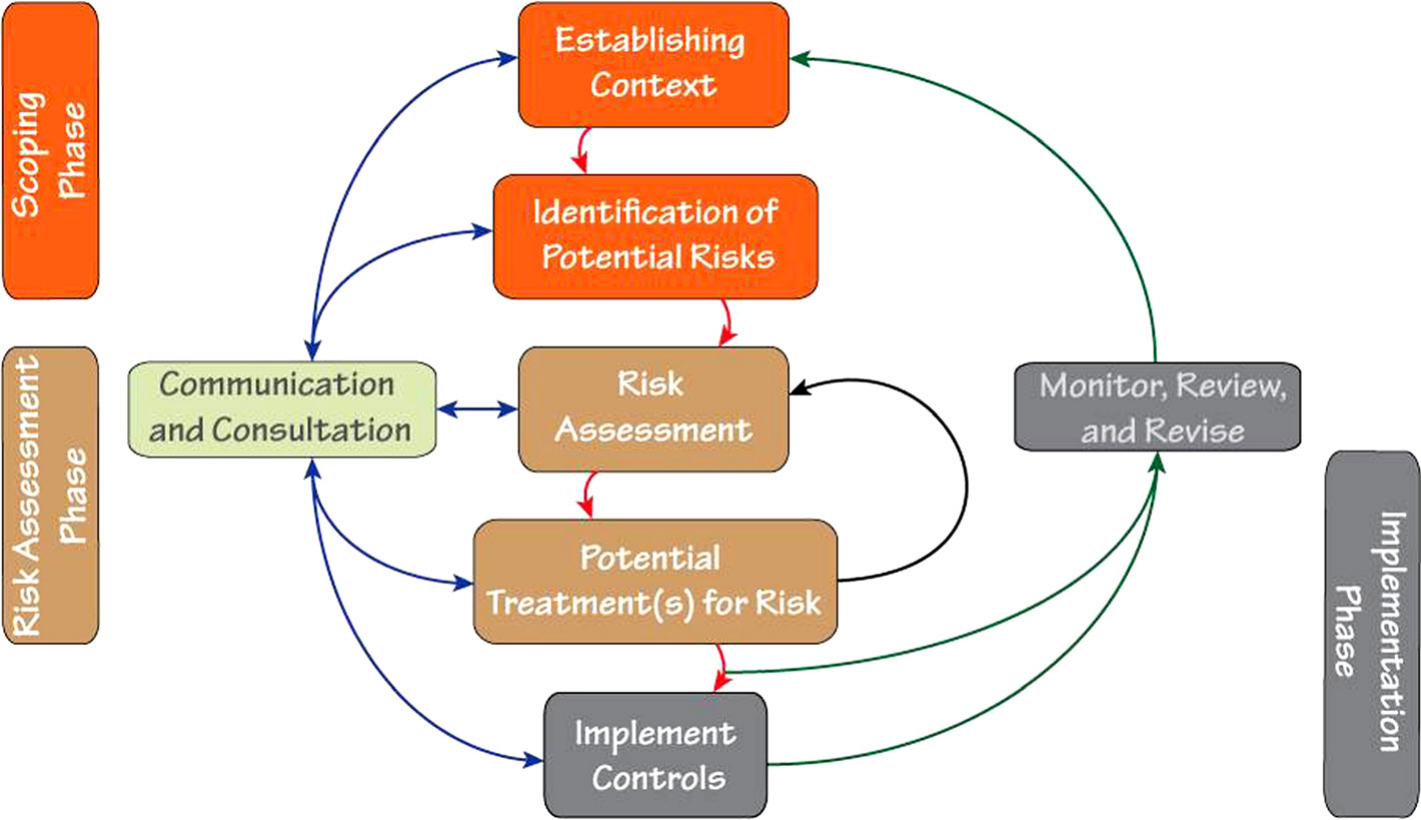
Risk management approach for occupational safety and health, adapted from the Minerals Industry Safety and Health Centre (MISHC), University of Queensland, Australia.

The RM approach (Figure 1) can be used at all levels of the management structure. It is a process that organizes information about an unwanted event in an efficient, orderly manner so that decision-makers can make informed choices. RM tools help focus attention and resources on the most significant risks. The approach is characterized by a cyclical pattern; as resources are focused on particular tasks and their associated risks, it is expected that the risk of an event will improve (i.e., the likelihood of an event resulting in injury is reduced). This results in a reorganization of the risk ranking, placing the emphasis of resources on identified priority areas.

Successful RM implementation includes a systematic, yet flexible, approach that invokes collaboration of team-based working groups (representing the full rank and spectrum of personnel) aimed at developing strategies to mitigate risk and avoid loss. The RM approach is one that can be applied both broadly and in specific areas, depending on overall objectives and the nature of the industry and potential hazards. It has been noted that the “primary responsibility for ensuring health and safety should lie with those who create risks and those who work with them” [5]. Managing the risks faced in hazardous occupations such as firefighting is meant to train decision-makers (which encompasses all employees to an extent) to objectively simplify the task (or event) at hand, consider risk in their decisions, and make logical choices without taking away their authority. Rather than develop prevention strategies solely from the top of an organization structure, this RM approach inherently requires direct input from those employees who are the focus of injury reduction interventions, as they are the individuals subject to the greatest risks. By engaging the employee through this approach, it is more likely to have: a feasible approach; buy-in from the employee; behavior change in relation to the safety culture and risk perception; and improved recognition and understanding of hazards and the risk they may pose [6,7].

### The fire service

Like many occupations with high physical demand and myriad hazards, injuries in the fire service are a regular concern for departments and, as recently reported, continue to be elevated [8], and in need of new strategies to aid in their prevention. Several studies have documented the increased hazards and risks associated with working within the fire service – from physiological stresses of fire suppression activities [9], and ergonomic and biomechanical loads [10,11] during patient assessments and transport, to psychological and post-traumatic stress disorders [12,13]. These diverse sources of hazards suggest that an adaptable approach to managing risks would be of benefit to the fire service. Although RM is an integral aspect of fire service operations, to our knowledge there are no published reports documenting the implementation of formalized RM in the fire service. We previously published an international comparison of fire service injuries showing that the United Kingdom (UK) fire brigade with the most advanced proactive RM program (legislatively required for over 20 years), also had injury rates well below those of fire departments in the US, Canada, and Australia [14], although it was not possible to fully adjust for fire service structural differences and approaches among countries.

In early 2010, the Tucson Fire Department (TFD) partnered with occupational health researchers to implement a RM program targeting the frequency and severity of injuries to their workforce. The primary aim of this research was to implement task-specific, risk-based intervention strategies within TFD, and to assess the efficacy of the approach. As previously noted, the RM program involves a participatory methodology, so that individuals with whom the interventions are aimed at supporting – in this case, firefighters and paramedics – are directly involved in the process. This manuscript documents how the RM approach was applied to three common activities or operations in the fire service: patient transport, fireground operations, and physical exercise. The study was approved by the University of Arizona Institutional Review Board, Tucson, Arizona.

### Risk management methods

#### Risk management model

The RM process consists of three phases: (1) scoping; (2) risk assessment; and (3) control implementation (Figure 1). A scoping phase begins the process by establishing the context of the operation or job task, understanding the potential hazards and identifying unwanted events. This generally includes a process mapping technique that visually represents the stepwise process for each group task, and a description of the typical activities and hazards within each step. Outputs from the scoping phase help inform specific risk assessment technique(s). For example, a risk assessment focused on preventing a specific injury-producing event (e.g., flashover, roof collapse) would likely utilize different techniques and instruments than an assessment of lower back injuries during a job-task (e.g., patient transport).

During the risk assessment phase, hazards are more formally detailed and understood so that a risk analysis may be completed. This risk analysis can produce qualitative, semi-quantitative, or quantitative measures, which attempt to provide information on the likelihood and consequence of a particular event or injury. Various instruments can be used to help analyze risk and will often vary based on the type of unwanted event(s), available data, and consideration of end-user goals. An example instrument, used in this study, was the Workplace Risk Assessment and Control (WRAC) form. The WRAC provides a semi-quantitative method for estimating risk that complements the process map generated in the scoping phase [1,15]. Using a consensus approach, potential hazards or exposures described in each step are (semi)quantified by agreeing to the likelihood and consequence of the particular hazard (or exposure), which corresponds to a pre-determined range of values within a risk matrix (example available in Additional file 1: Figures S1). In the case of a 4x4 matrix, the likelihood of exposure to a hazard is scaled to represent: (1) unlikely (<10% probability of occurring within the last year), (2) possible (10-30%), (3) likely (30-75%), and (4) almost certain (>75%). Similarly, consequences (or the hazard effect) are scaled to reflect: (1) minor effects (e.g., first aid cases), (2) moderate (e.g., lost time injury), (3) major (e.g., loss in quality of life), and (4) maximal (e.g., single/multiple fatalities). Risks are then characterized describing the extent and severity of risk to individuals and the overall workforce. This characterization also includes an evaluation of the overall quality of the assessment with a description of uncertainty. RM tools thereby help focus attention and resources on the most significant risks.

Potential new or adapted control strategies are then considered for mitigating the identified risk(s). Once these control strategy concepts are finalized and approved, the implementation phase is initiated, which actively develops and applies the new controls (or modifications of existing controls) after ensuring the greatest risk(s) have appropriate levels of control measures in place. A regular review and process evaluation is also instituted to assess overall effectiveness and to ensure that no new unintended risks are introduced. Active revision and fine-tuning of the control strategies are made as deemed necessary.

#### Task-based Application of RM

The majority (60%) of injuries occur in the TFD population during patient transport (16.9%), fireground (10.2%) and physical exercise (32.9%) activities and operations [8]. The objectives during the first year of the study were to identify, analyze, and characterize the hazards and risks associated with injuries during these specific work processes. Three cross-sectional teams of 6-10 individuals – from the newly commissioned to upper management – were recruited to participate in the study. Special emphasis was placed on involving firefighters, medics, engineers, captains and union representatives that were active in the field and exposed to the most injury risk. Select fire station crews were recruited to ensure distribution of employee ranks and also to represent geographic aspects of the city. For example, some sections had a higher proportion of emergency responses specific to industrial incidents and structure fires, whereas others served a larger elderly population and consequently had more frequent medical responses.

Over the span of approximately eight months, each team completed the scoping and risk assessment phases of the RM model, leading up to the implementation of control strategies. Six sessions were held approximately 4-6 weeks apart, lasting no more than two hours (of their 24-hour shift), and were held at the respective crew’s fire station while on-duty. All three focus areas (patient transport, fireground and physical exercise) were addressed concurrently. The first two sessions were dedicated to the scoping phase and mapping process, while the third and fourth sessions focused on the risk assessment. The final two sessions characterized risk, reviewed control strategies currently in existence, and identifying potentially new controls.

The nature of patient transport and fireground operations allowed for linear, though multifaceted, decision-making steps to be detailed, enabling the identification of hazards and characterization of risks. Characterizing the risks for physical exercise was difficult to frame in a systematic manner, due most notably to inconsistencies and wide variations between individual and group workouts, as well as differences between maintaining “fitness” and “fit for duty” status. In consultation with fire department senior personnel, an alternative (and simplified) approach was devised and implemented to the physical exercise group, focusing on a more open dialogue aimed at describing what exercise in the fire service is meant to achieve, how it is currently performed (and the limitations therein), and how to make progress toward the ideal situation. Once understood in this context, areas for improvement were identified and controls suggested.

For all three groups, risks for injury were not isolated in a particular segment of the individual processes, but rather spread throughout. New control strategies, as well as modifications to existing controls, that had the potential to mitigate both the likelihood and consequences of the risks were identified. Selecting which control strategies would be developed and implemented during the research time period was aided by using aspects of Runyan’s third dimension to the Haddon’s Matrix to help select between multiple controls solutions [16]. Specifically, individual crews, department administrators (who better understood costs) and researchers ranked control strategies according to five criteria: effectiveness, feasibility, cost feasibility, sustainability, and the potential for unintended risk; each with pre-defined explanations. Each criteria was assigned a simple priority ranking between 1 (lowest priority) to 5 (highest priority) and summed among the participants. An additional four months were necessary for reviewing and selecting controls for implementation. The implemented control strategies were selected collaboratively between researchers and department leadership after consideration of priority scores, estimated department resources and costs, and the likelihood to develop, implement and evaluate controls within the timeframe of the study.

### Results of the risk management process

#### Implementing the risk management process

At the time of the first study session (baseline), 25 commissioned employees were consented to participate in the focus groups. However, others joined throughout the scoping and risk assessment phases, for a total of 34 participants (patient transport n = 12, fireground n = 12, and physical exercise n = 10). Recalling that these sessions were held while on-duty, approximately 64% of individuals that were eligible (i.e., consented and assigned to the relevant participatory group) to participate were in attendance, on average. Participation in the individual sessions ranged between 38% of those eligible to full (100%) attendance, with absences due to sick leave, injury, furlough, rotation to another fire station, or vacation. Table 1 includes baseline demographic data for consented participants, which resembled the workforce population as a whole.

**Table 1:** **Baseline participant demographics (n = 25)**

**Demographic**	**Value**
Task Group	
Patient Transport	36%
Fireground	32%
Physical Exercise	32%
Male	92%
Age (years)	Mean 39
	Range 24-53
Self-reported race or ethnicity	
White, non-Hispanic	80%
Hispanic	12%
Other	4%
Missing	4%
Rank	
Deputy Chief	4%
Captain	24%
Engineer	12%
Firefighter	28%
Paramedic	28%
Inspector	4%
Time in current rank (years)	Median 4
	*IQR 9

* IQR = interquartile range.

#### Process mapping

**Figure 2:**
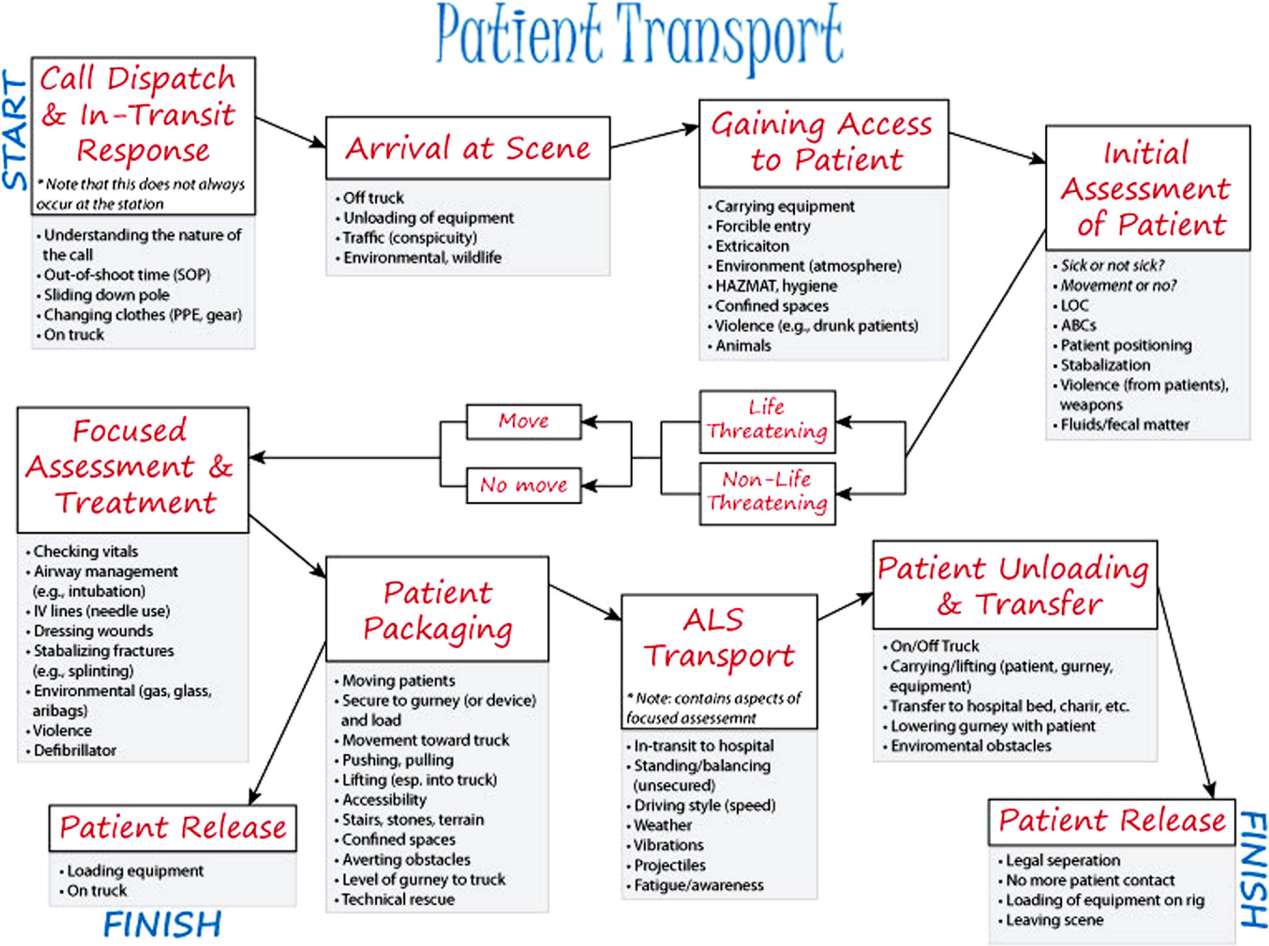
The patient transport stepwise process, along with general activities and hazards (listed in grey boxes).

The goal of the scoping phase was to understand potential hazards and identify the unwanted events (i.e., injuries), as they pertain to each task. One of the more valuable products generated from this phase was the individual process maps. As illustrated in Figure 2, the progressive steps of the patient transport process are documented along with their respective activities and potential hazards (see Additional file 1: Figures S2 and S3 for the fireground and physical exercise approach).

#### Identification and selection of control strategies

Among the three task groups, 45 potential new or modified control strategies were identified from the risk assessment phases (Table 2). Forty-seven percent (n = 21) of controls were educational in nature, while 33% (n = 15) entailed some type of enforcement. The remaining controls pertained to engineering solutions (n = 7) and incentives (n = 2).

**Table 2:** **Identified control strategies from participatory scoping and risk assessment phases**

**Control strategy**	**Priority rating**
**Patient Transport**	
Patient transport module for probationary firefighters	112 ‼
Apparatus placement for all scenes	109
Review police request protocol	106
Standardized in-station call review among crew	103
Continuing education for patient packaging and lifting	100
Establish chest compression rotation procedure during CPR	100 ‼
Improved communication for patient lifting assistance	100
Review Clawson questions and format	97.5
Emphasizing and improving fitness training	96.5
Distribution of techniques for heaving patient lifting	96
Station checklist and inventory for all equipment	94.5
Testing patient transfer devices	91 ‼
Heart Saver and zone dispatching systems	88
Continuing education for airbag deployment	87.5
Combative Patients Training	84
Investigate gurney design options	82
***Physical Exercise***	
Update and revise exercise SOP	105 ‼
Better defined fitness standards and levels	104
Mandatory workouts each workday	103
Improved access to exercise information	101
Improve structure and monitoring of 8-hour employees	101
Explore new cadet point system for physical fitness	100
Improve station exercise equipment and facilities	97 ‼
Increased role of Peer Fitness Trainers	96 ‼
Top-down advocacy of priorities	95
Periodic “fitness checks” during the year	95
Reinforce fitness progress and achievements	94
Incentives for exercise adherence	91
Structured exercise sessions	89
***Fireground Operations***	
On-shift safety critiques about selected calls	71
Enforce PPE use during demobilization and cleanup	70 ‼*
Enforcement through disciplinary matrix	69
Improve rehab adherence	67 ‼†
Improve rehab protocols and details	67 ‼†
Peer safety check before post-suppression activities	67 ‼*
Visual reminders for health and safety	66 ‼
Prohibit cell phone use while in-transit to call	66
Increase emphasis on maintaining fitness	65
Improve access to health and safety information	64
Increased CEs and trainings for Captains	64
Rewards and incentives to improve compliance	63
Communicate individual health status during lineup	60
Improved utilization of 0700 “Wake up” call	56
Collaborate with industry partners	54
Improved communication and personnel tracking tech	51

‼ Selected controls for implementation during study period.

*† While individually identified by the group participants, these controls were combined.

Nine controls strategies were selected for implementation during the study period (identified by “‼” in Table 2). The selected control strategies for patient transport were primarily focused on ergonomic issues and reducing the risk associated with both acute and cumulative increased loads throughout the process. Recruit training learning modules concerning patient moving and lifting were restructured; ergonomically-designed slide-boards for lateral transfers were outfitted on all ambulance gurneys; and all crews were trained on new recommendations to rotate chest compression responsibilities during cardiopulmonary resuscitation (CPR) procedures.

Fireground control strategies pertained mainly to improving situational awareness and the reinforcement of safety protocols from peers. It was suggested that individual-level situational awareness may be depressed during demobilization and clean-up steps of fireground response, in contrast to the heightened awareness during active fire suppression or rescue scenarios. As a result, and in addition to general fatigue, the awareness of surroundings and motivation to re-don personal protective equipment (PPE) was considered to be lowered and responsible for a number of minor and moderate injuries. The addition of improved rehab protocols (a rest from active fire suppression, during which time body temperatures decline and vital signs normalize) also support the ability to take appropriate precautions. Aside from fire suppression, visual reminders were also recommended to prevent common injuries. Specifically, health and safety reminders were designed for hydration awareness (placed in station restrooms), 3-points of contact reminders when getting on or off apparatus, and wearing of appropriate PPE around station hose towers, and chemical cabinets.

For physical exercise, it was acknowledged that each individual exercises with different goals in mind (e.g., improving strength versus cardiovascular health). Since all participants understood the physical nature of the job and the need to maintain one’s health and fitness levels to meet the demands of job tasks, banning exercise activities all together, as had been reported in other fire departments, was not considered a viable option. However, the lack of structure, education and oversight in the practice of daily exercise activities was recognized as both a significant hazard and opportunity. As a result, the alternative scoping and risk assessment approaches used for physical exercise produced a series of control strategies focused on improving the structure and management of on-duty physical exercise, including (1) updating physical exercise equipment and facilities across stations, (2) increasing the roles and responsibilities of certified peer fitness trainers (PFTs), and (3) updating the department’s fitness and exercises protocol.

#### Safety committee

Another positive outcome of the scoping and risk assessment phases was the re-establishment of an internal department safety committee that, similar to the makeup of the participant groups, is comprised of a cross-section of department employees (both civilian and commissioned). This committee was charged to assist in the implementation and evaluation of the most relevant control strategies identified through the project (i.e., the third phase), in addition to continuing to identify and implement proactive and relevant improvements to the health and safety of all department personnel that were previously distributed among various other operational committees. This new group helped focus and centralize department-wide awareness and efforts regarding health and safety, while satisfying the National Fire Protection Association (NFPA) 1500 standard for occupational safety and health programs. Members were asked to voluntarily serve for two years, with the potential to continue additional years to maintain both continuity of safety efforts and fresh perspectives from newer (rotating) committee members.

### Process evaluation methods

#### Process evaluation

Qualitative data were collected to document the implementation of the RM process, and better understand which aspects worked well and which could be improved. A member of the study team (KP) with expertise in qualitative research methods conducted focus groups with each of the three operation groups after the scoping and risk assessment steps. Also, a total of 4 key informant interviews with fire department leadership and two focus groups with 8 firefighters were conducted after implementation of control strategies. The qualitative data collected after scoping sessions (October 2010, 1 month after scoping and risk assessment phases) explored the perceptions of the RM process, specifically: the scoping sessions; implementation of the RM process; and perceived role of the RM process for reducing firefighter injuries. Participants in the focus groups after the scoping and risk assessment steps included individuals who were initially consented and participated in some aspect of the RM process.

Data were collected from key informant interviews with fire service leadership and a focus group with firefighters after implementation of control strategies (June 2013, 3.5 years after beginning of RM process). These data collection efforts again explored perceptions of the RM process and its perceived impact on injury, facilitators and barriers of implementation, sustainability of the control strategies, and recommendations for replication. A brief, self-administered, anonymous, demographic questionnaire was also administered prior to the start of the focus group. With participants’ permission, sessions were digitally recorded and transcribed by a professional transcription service. Focus groups and interview instruments can be made available on request to the corresponding author.

#### Qualitative analysis

Initially, transcripts were validated by comparison against each corresponding recording. Next, the validated transcripts were read repeatedly, in addition to notes from the scoping sessions. One of the investigators (KP) led the analysis using an open coding process followed by topic coding to identify and explore the content of the dialogue on recurring topics across the operation groups and identify key themes [17,18].

### Process evaluation results

#### Perceptions of the risk management process

Results of the baseline survey from the 25 participants who were present for the first scoping session are presented in Table 3. Ninety-two percent of the participants felt that all firefighting injuries are not preventable; however this view changed dramatically when directly asked about specific tasks, as 76% believed task-specific injuries could be prevented. Most firefighters (72%) felt they solely were responsible for their own injury risk, whereas 8% felt they shared this responsibility with their Captain or Chief, and 20% felt that everyone shared the responsibility. Nearly half (44%) answered that they alone had the responsibility for managing their injury risk, 4% said their Captain or Chief, 36% said responsibility should be shared with their Captain or Chief, and 20% answered everyone.

**Table 3:** **Baseline perceptions of injury among session participants (N = 25)**

**Baseline perception of injury**	**Yes (%)**	**No (%)**	**Both (%)**
Do you believe that all injuries during firefighting are preventable?	8	92	--
Do you believe that getting injured during firefighting is “part of the job”	28	68	4
Do you believe that injuries during a (specific to each group - patient transport, fireground, or physical exercise and training) can be prevented?	76	16	8
Do you believe that you have control over your own risk of sustaining an injury while working as a firefighter?	80	12	8

The themes from the qualitative data can be summarized according to three main areas: 1) perceptions of the RM process, 2) perspectives of the scoping process, and 3) perceived effect of the RM process on firefighter injury prevention.

#### Perceptions of the RM process

The overwhelming majority of participating firefighters emphasized the value of the RM process. They praised the participatory bottom-up approach, which many felt was the first time they were able to provide input prior to implementing a workplace intervention. Several firefighters talked about how the potential control strategies came “straight from us,” and contrasted the process of providing input during the RM process to prior instances when controls were implemented without being previously discussed. The overwhelming perception was that speaking with the firefighters about the risks encountered helped to validate the process and lead to more accurate solutions. This perspective was captured well by one participating firefighter who said: “…that is one of the advantages of the [RM] process, because they’re actually going to the people who are in the trenches, doing the work, instead of forming their own opinion of what we would see as being a problem, they’re actually talking to people who are going to be experiencing some of these problems, and so it makes it more accurate, I believe.”

Many firefighters also spoke about the benefits of having a diverse group of firefighters, across ranks and positions, involved in the process. Many believed that doing so emphasized the bottom-up approach. For example, several firefighters noted that if the RM process only involved Captains, it would have been perceived as a top-down approach and not very participatory.

#### Perspectives on the scoping process

Nearly all of the firefighters spoke about the importance of having organized scoping sessions. While some admitted to not always looking over the materials closely in advance of the scoping sessions, they did appreciate that the materials were provided in advance and that everyone knew what the scoping session was going to cover. The credibility of the leader running the scoping sessions – an individual who was an academic, but also very knowledgeable about the fire service and process – was also discussed by many participants.

Walking through each step of the operation or activity during the scoping process was viewed as helpful to illuminating the specific ways that an injury could occur. Several firefighters spoke about the utility of “breaking down” each step and thinking about the risks associated with each task. Many felt that the process allowed them to think through the steps and discuss each step, which was very useful in thinking about how an injury could occur.

While the scoping sessions were seen as valuable and clear, especially for patient transport and fireground, some specific challenges were raised regarding the physical exercise scoping process, which was seen as too broad at first because it also included drilling (i.e., firefighter drills). The firefighters who participated in this specific group felt that applying the RM process to physical exercise was challenging because these activities were too nebulous. Once the scope was narrowed and drilling was removed, physical exercise, while still a detailed operation, was viewed as more manageable.

Another commonly described challenge was the inconsistency in attendance for each of the scoping sessions, due to the sick leave, vacations, etc. Some firefighters described how having to bring a participant up to speed, after he or she missed a session, was somewhat disruptive and not a very good use of time. It is believed that attendance would have improved if sessions were held off-duty and away from the fire station, ideally with some sort of compensation.

Some firefighters mentioned how finding time to participate in the scoping process was a challenge. Many firefighters stated how they would have preferred receiving overtime for participating in the scoping sessions, rather than being taken out of service. Several of the participating firefighters spoke about how being out of service may have affected the other crew members, because “while we’re out of service, they’re on the calls.” Firefighters did not know if their fellow firefighters were upset that they were taken out of service and they noted that they had not heard that they were; however, a few firefighters shared that it is possible that some firefighters may have resented that they were out of service because of the research.

#### Perceived effect of risk management on injury prevention

A common theme that emerged across all groups when asked about the effect of the RM process for injury prevention was that it was too soon to know what the impact of the RM process would ultimately be. In the words of one participating firefighter, “…true assessment of value of risk management will be seen once strategies are implemented and impact assessed.” The control strategies that were developed were noted as needing to be enforced in order to reduce risk. Firefighters also spoke of the importance of changing the fire service culture, as well as needing the captains to support and reinforce the changes as ways to reduce injury. Finally, in terms of sustaining the control strategies, firefighters also discussed holding each other accountable. One firefighter expressed this perspective well when he said that, “firefighters need to support each other and figure out how to get those not doing to do.”

When asked about the impact of the RM process on injury, many firefighters expressed that the effect of each control strategy on injuries hinges on how the policies and practices are enforced. In addition, some firefighters spoke about how simply participating in the scoping process helped them think differently about injury risk. For example, the scoping process involved sharing injury data, which was used to inform the WRAC process. Several of the participating firefighters spoke about value of seeing the circumstances of the injury, which were presented in an easy to understand and clear way, to better understand the burden of a particular injury. In the words of one firefighter: “…I got to look at the injuries [and read the narratives] and go well, that guy wouldn’t have lacerated his hand, if he had his gloves on, so I think about the stuff that I do…that was probably one of the bigger things for me, was just realizing that a lot of people are getting their minor injuries that could have been prevented. So simple.”

#### Reflections on the risk management process

After all control strategies were actively implemented (3 years after the study began), firefighters still felt that the RM was very valuable, with all of them stating they were pleased that they participated in what one firefighter called “groundbreaking” research for the fire service. Firefighters who agreed with this perspective stated that implementing RM in the fire service was groundbreaking because it was the first time the department took a systematic approach to exploring injury risk and involved the firefighters – they felt “listened to.”

Many firefighters stated that the systematic approach supported by the RM process is one that is sustainable. The approach to looking at risks – one that breakdown tasks – allowed firefighters to have input on elements that they “have always thought would make a difference.” For example, since the firefighters were involved in identifying the control strategies, they were able to suggest strategies that they felt would make a difference. Several firefighters noted that the evaluation research being conducted will not only validate their perceptions about what works, but also will provide evidence for the department to continue to support effective control strategies.

Nearly all of the firefighters spoke about the sustainability of the control strategies, which for some made the long term goal of the study (i.e., implementing the RM process) unclear. Some participants felt that while the RM process identified many possible control strategies, only some (20%) were implemented. Some firefighters who participated in the RM process wondered if the strategies not selected for implementation during this study period would eventually be implemented. One example that was shared involved the new policy for fireground rehabilitation that emerged from the RM process. It was shared that the new rehabilitation process might affect resources, namely personnel, since firefighters are now required to rehab, which could potentially require additional crew members during a fire response to take the place of the firefighters who go to rehab. Some firefighters felt that these costs were not discussed during the RM process, and it was particularly important because of changes in the size of the department and budget constraints. The same concern was raised for the control strategy involving the use of peer fitness trainers (PFTs). Resources are needed to train the PFTs and ensure they have appropriate facilities and equipment to effectively promote fitness and optimal health among firefighters. Equipment was purchased with funds from the grant that supported this work, but many questioned what would happen when resources were needed to maintain the equipment. All of the firefighters involved in this discussion felt that if a commitment is made to a control strategy, there needs to be an equal commitment to providing resources and funding. A few firefighters cautioned how perceptions about resources could be a barrier: if firefighters felt that the administration was not committed to allocating resources to implement control strategies, firefighters might not buy-in to the use of the RM process to reduce injuries.

At the end of the RM process, essentially all of the firefighters viewed it as positive; however, the RM process raised more issues as a whole, and in the words of one firefighter, “opened up other windows of things to consider.” It was clear that while firefighters felt that addressing these other issues would be challenging, having these discussions that involve firefighters about how best to promote firefighter safety and health is a good thing for the service in the long run. Table 4 presents recommendations and considerations from the study participants as part of the process evaluation, and is intended to guide replication of the RM program in other fire departments.

**Table 4:** **Recommendations for replicating the RM process in the fire service**

**Recommendations**	**Considerations and suggestions**
1. Planning or organizing for the scoping sessions	- Involve a diverse group of firefighters across rank and experience
	- Think about how to get people to participate (pay overtime vs. take out of service during one’s shift)
	- Utilize best practices for effective participatory engagement that bolsters the “bottom up” approach
	- Choose a strong facilitator to lead the scoping sessions
	- Recognize that not everyone will show up for every session
2. Identify clear tasks for the RM process	- Select task with definable steps to facilitate mapping process
	- Have clear tasks to help identify specific control strategies
3. Utilize quality data	- Use department level data (and station level if possible)
	- Present data clearly and by task
4. Understand culture	- Consider elements of firefighter culture. Utilizing a “bottom up” approach does not always lead to buy-in from firefighters in the field (beyond supervisors) not involved in the process.
	- Some FF may not support the RM process because it is not “how they always do it”
5. Recognize the importance of technical assistance (TA)	- Ensure technical assistance in compiling data for the RM process
	- Provide technical assistance for other aspects of the RM process, such as the mapping and ranking of control strategies
6. Understand available resources	- Firefighters will not buy-in if they feel that the administration is not willing to invest resources
	- Highlight the cost savings of RM to support financial investment in control strategies
7. Provide regular communication	- Communicate expectations of the RM process upfront, including resources available for control strategies
	- Provide regular communication to all firefighters since those not involved in RM may not be aware of all of the activities

## Discussion

One of the primary objectives for the study was to implement a RM process that could be sustained by the fire department, thereby serving as an exemplar to other departments. The RM model utilized in this study represents a systematic approach aimed at improving decision-making in the workplace, which was widely supported by the fire department participants. While approaches to RM can vary greatly across organizations, the core components of the RM process (scoping, risk assessment, and the identification and implementation of control interventions) were completed in this study. The inclusion of all ranks in the RM teams, a common but not universal RM approach, was clearly identified as beneficial by the study participants. Variations on the RM approach are largely dependent on the fire department setting, organizational structure and available resources. For example, during a visit by the study investigators to the UK to inquire about their RM process, a variety of approaches to RM were noted including having civilian RM experts direct the process, working with uniformed firefighter colleagues; having RM teams comprised primarily of upper ranks of firefighter administrators; and having all individual firefighters trained to perform at least one RM evaluation. Each approach was suitable for the individual department context, thus fire departments considering implementing a RM process should determine which adaptations are needed to successfully implement the RM process. For instance, in the UK, fire departments undertook the RM process based on government regulation, while the process undertaken by TFD was entirely voluntary and without knowledge of long-term funding or support mechanisms in place.

One of the recommendation categories for replicating the RM process (Table 4) pertained to the planning and organization of scoping sessions. Conducting the scoping sessions requires ample time toward the planning process and preparation of materials before the RM process begins and between each session. When this approach was first introduced in the Australian coal mining industry, the scoping phase alone took, on average, two years. As described in the methods, the scoping and risk assessment phases combined were completed over the span of eight months in the current study. During this preparation time, the facilitator of the scoping sessions should focus on collecting and interpreting health and safety surveillance data, meeting with key personnel, and conducting field evaluations to best contextualize the risks discussed and appreciate the organization’s culture and perspectives on health and safety. The importance of these preparatory activities cannot be overstated, and should lead to improved involvement and buy-in once control strategies are identified and implemented. In addition, the RM process benefits from the facilitator’s knowledge of the various risk assessment approaches and instruments that can be applied during the risk assessment phase of the RM process, including (but not limited to) Haddon’s Matrix [16,19], fault-tree analysis, hazard and operability (HAZOP) analysis, bowtie analysis, WRAC (used in this study), or any other tool that attempts to list conceptualize etiologic factors for injury and to identify potential preventive [1,15,20].

There are a number of limitations to the RM process carried out in this study. The RM process within TFD was undertaken with financial support limited to the duration of the grant’s funding cycle and was supportedby a team of academic researchers. Not every fire department has a close partnership with academia, thus the generalizability of these findings is limited in regards to replication within the fire service. Many RM processes are focused on critical controls designed to focus on the most catastrophic (and potentially fatal) hazards. In the current study, however, prevention efforts were focused on hazards resulting in frequent injuries and not just those that were life threatening. While the current study focused on interventions generally specific to one task or operation, other RM approaches can help reduce the risk from single, catastrophic events (e.g., flashovers, roof collapse, etc.), or even specific injury types, such as back sprains resulting from the lifting and transfer of patients. A benefit of RM is that each organization can choose approaches that the membership believes will yield the greatest benefit. In the UK, RM is a mandated component of fire service policies and practices, and is often permeated throughout the ranks. The TFD RM process could eventually reach this level of incorporation into policies and practices and distribution throughout all firefighters, but necessity dictated that the initial focus be limited to specific activities and a smaller group of participants. The timing of this study also took place during the US recession of 2007-2009 when cities and municipalities were undergoing significant budget challenges, which limited the number of RM controls that could be implemented. Finally, firefighters were not randomly selected to participate in the RM process, therefore raising questions regarding potential selection bias. Although a diverse sample of firefighters was sought to participate in the RM process, firefighters that responded to a large number of fires were selected for the fireground group; stations with a high volume of medical calls for the patient transfer group, and so on. While acknowledging that intentional selection of fire stations was a component of this project, this strategy is believed to have enhanced the project, since individuals with high exposure and experience to certain tasks were recruited for the RM process.

## Conclusion

The RM process implemented within the TFD targeted many significant injury hazards in a manner that was looked upon favorably by most firefighters, based on the qualitative data collected. Firefighter injuries remain a significant and preventable public health problem, and RM is one approach that with strong implementation has the potential to reduce the risk of injury. As RM becomes a more widely accepted process in the fire service (and other occupational settings), additional study of the effectiveness of different approaches and control interventions will help guide this process and serve as models to those disciplines.

## Additional file

Additional file 1:
**Figure S1.** 4x4 risk matrix for (semi)quantifying risk from hazard exposures. **Figure S2.** The fireground stepwise process, excluding general activities and hazards. **Figure S3.** The alternative and informal physical exercise approach.
