# Supplementary material for: Establishing a proactive safety and health risk management system in the fire service
Source: BMC Public Health. 2015 Apr 19;15:407. doi: 10.1186/s12889-015-1675-8 (PMC4409742; doi:10.1186/s12889-015-1675-8)
Supplement: Additional file 1: — Figure S1. 4x4 risk matrix for (semi)quantifying risk from hazard exposures. Figure S2. The fireground stepwise process, excluding general activities and hazards. Figure S3. The alternative and informal physical exercise approach. [file 12889_2015_1675_MOESM1_ESM.docx]

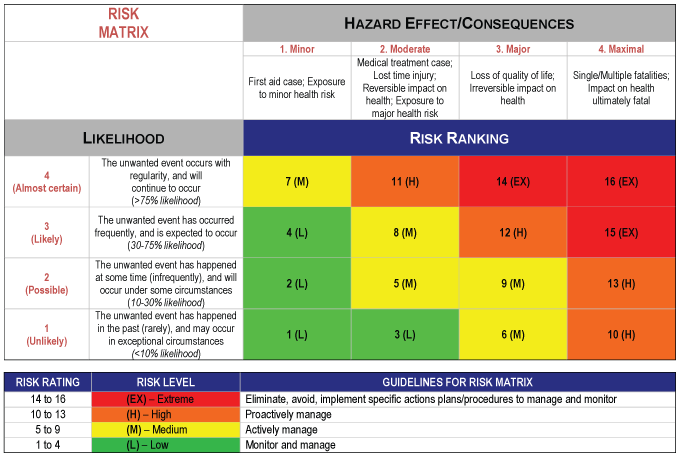


Supplemental Figure 1. 4x4 risk matrix for (semi)quantifying risk from hazard exposures


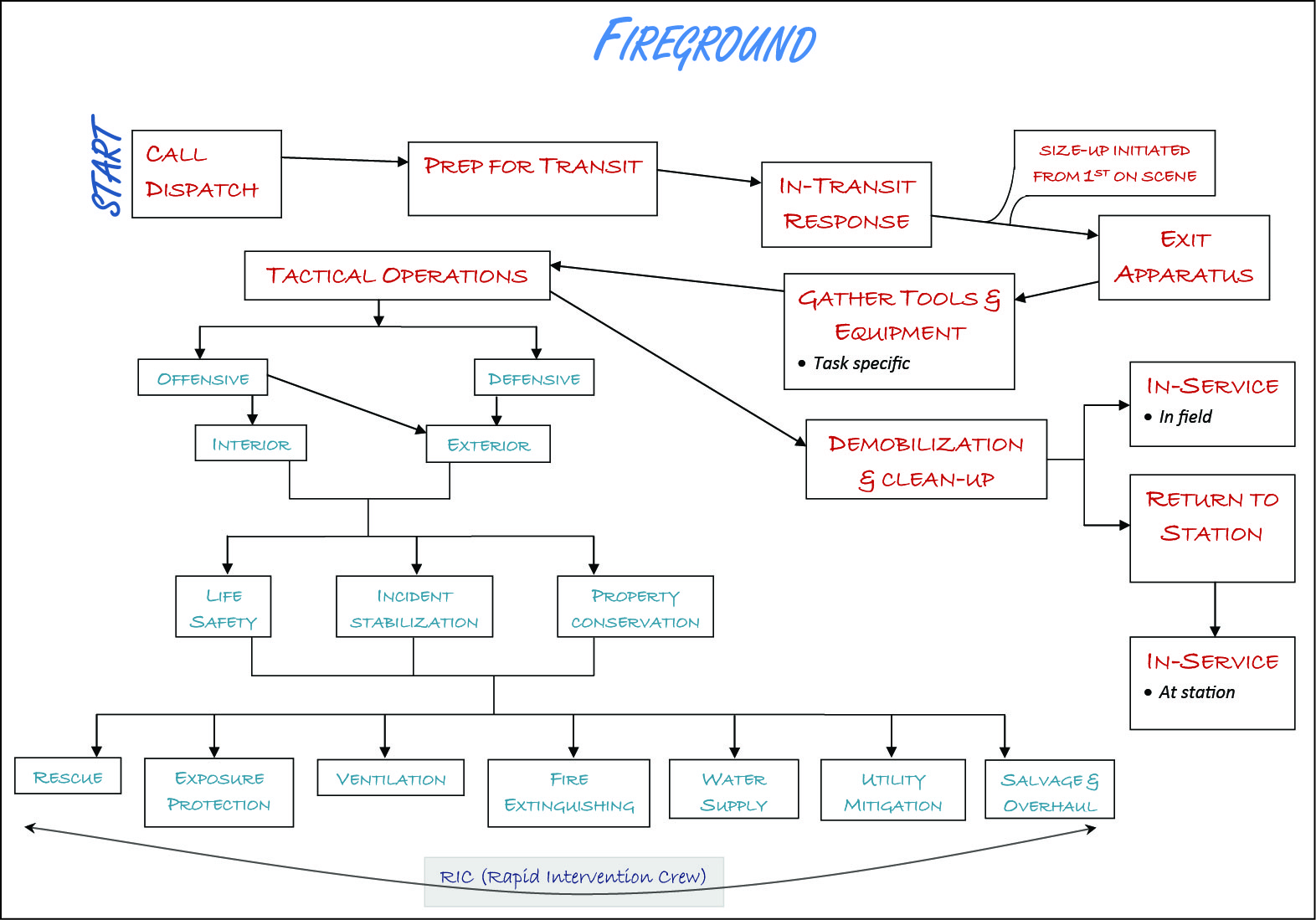
Supplemental Figure 2. The fireground stepwise process, excluding general activities and hazards


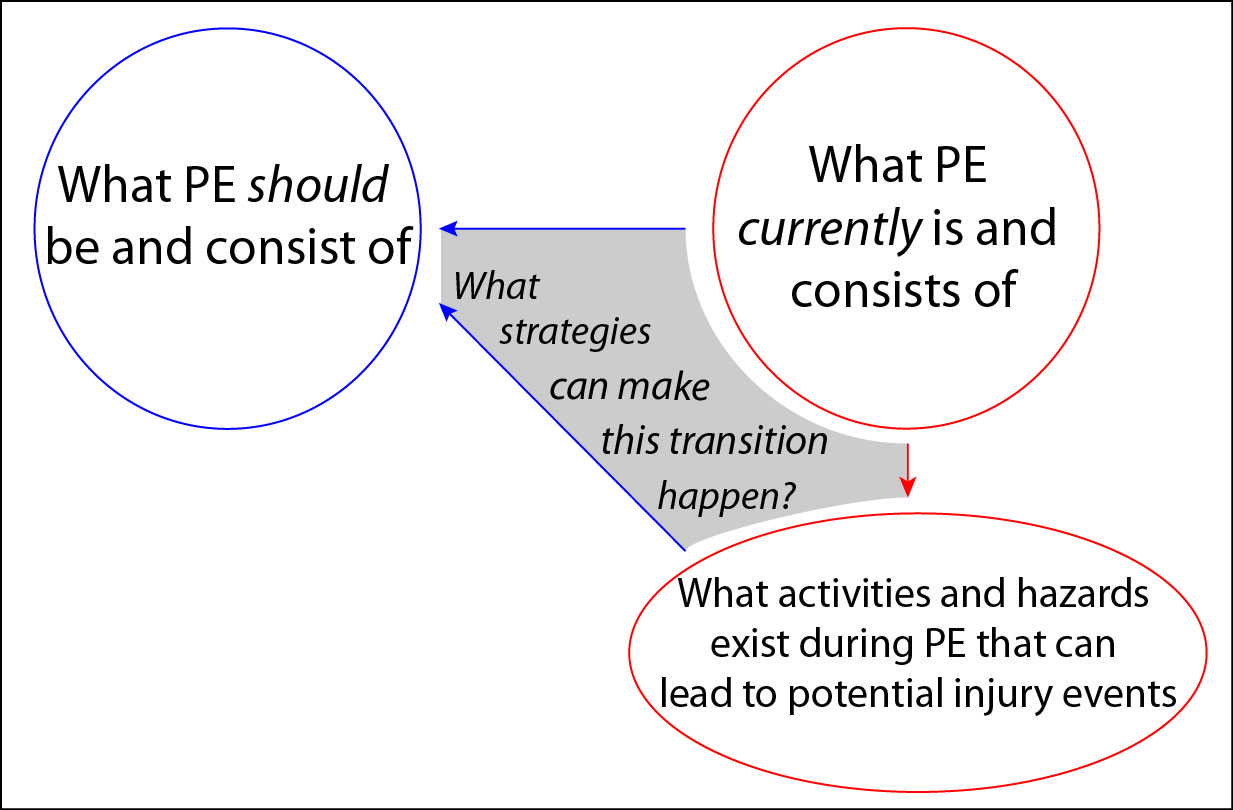
Supplemental Figure 3. The alternative and informal physical exercise approach
